# Evaluation of postoperative pain after endodontic treatment in molars with and without foraminal enlargement: a prospective randomized clinical trial

**DOI:** 10.1038/s41405-026-00412-5

**Published:** 2026-03-19

**Authors:** Fabiana Menezes Galdino de Aragão, Carlos Eduardo da Silveira Bueno, Rina Andrea Pelegrine, Daniel Guimarães Pedro Rocha, Carlos Eduardo Fontana, Vini Mehta, Wayne Martins Nascimento, Ana Grasiela da Silva Limoeiro, Marilia Fagury Videira Marceliano-Alves, Alexandre Sigrist De Martin

**Affiliations:** 1 Faculdade São Leopoldo Mandic, Instituto de Pesquisa São Leopoldo Mandic, Campinas, São Paulo Brazil; 2 Pontifícia Universidade Católica de Campinas, Departamento de Endodontia, Escola de Ciências da Vida, Campinas, São Paulo Brazil; 3 Department of Dental Research Cell, Dr. D. Y. Patil Dental College and Hospital, Dr. D. Y. Patil Vidyapeeth (Deemed to be University), Pune, India; 4 Faculty of Dentistry, University of Ibn al-Nafis for Medical Sciences, Sana’a, Yemen; 5 Department of Dentistry, Endodontics and Dental Materials, Bauru Dental School, University of São Paulo, Bauru, Brazil; 6 Postgraduate Program in Dentistry, Iguaçu University, Nova Iguaçu, RJ Brazil; 7 Department of Endodontics, Maurício de Nassau University Centre (UNINASSAU), Rio de Janeiro, Brazil

**Keywords:** Endodontics, Dentistry

## Abstract

**Background:**

Postoperative pain following endodontic treatment is a significant and common issue in daily clinical practice, frequently studied, and a source of concern for both patients and clinicians.

**Aim:**

This study aimed to evaluate postoperative pain and analgesic use after single-visit endodontic treatment with a reciprocating system with or without foraminal enlargement in mandibular molars with necrosis and apical periodontitis.

**Methods:**

Sixty patients undergoing endodontic treatment on mandibular molars were divided into two groups (*n* = 30): with foraminal enlargement (FE), with the working length set at 0.0 mm from the apex, and the other group without foraminal enlargement (WFE) at 1.0 mm short of the apex. Instrumentation was performed with the Wave One Gold System in a single-visit, rinsed with 2.5% sodium hypochlorite, and filled with a single cone and AH -Plus sealer. The patients were requested to rate their pain at 24, 48, 72 h, and 1 week on a visual analog scale (VAS) as nonexistent, mild, moderate, or severe, as well as to indicate the need for oral analgesics. The number of participants reporting pain was similar at both 24 and 48 h (*p* > 0.05).

**Results:**

Mean VAS scores were low in both groups at all time points, with no clinically meaningful between-group differences. At 72 h and 7 days, no participant in either experimental group reported pain. There was no statistically significant difference in the painkiller tablets taken between the experimental groups at any evaluated period.

**Conclusion:**

The frequency of postoperative pain and the number of analgesics taken by the patients were similar in the two experimental groups.

## Introduction

Pain after endodontic treatment is an important and common problem in daily clinical practice and the subject of several studies, and a cause of concern for patients and clinicians [1]. The occurrence of postoperative pain is usually due to an acute inflammatory response, a natural immune reaction that occurs when the body’s defense system responds to irritation or trauma experienced during the procedure, in the periradicular tissues, which can be associated with bacterial debris and extrusion, mechanical trauma, and chemical irritants [2].

One situation that may be related to mechanical trauma is intentional foramen enlargement (FE), which may be necessary from a microbiological perspective to reduce the microbial load during endodontic infection, as it may extend beyond the limits of apical constriction [3], and may affect the healing of chronic periapical lesions and the repair of periapical tissues [4].

Despite the proposed biological advantage, FE may lead to a higher incidence of postoperative pain due to direct mechanical irritation of the periapical tissues and/or extrusion of dentin debris during root canal preparation [5]. However, postoperative pain and the need for analgesic medication have been associated with techniques with and without foraminal enlargement (WFE) [6]. FE may lead to a higher incidence of pain due to mechanical irritation of the periapical tissues [7]. In addition, enlargement of the apical constriction can lead to significant extrusion of debris, resulting in periapical inflammation [5], postoperative pain [8].

Another problem associated with FE in the literature is the effect on the morphology of the apical foramen [9, 10], root canal transport, and the centering index of the preparation at the apical constriction [11]. Furthermore, no study to date has demonstrated the efficacy of intentional foraminal enlargement in facilitating instrument contact along the entire length of the root canal walls at the apical foraminal border. A recent study using micro-computed tomography concluded that foraminal enlargement resulted in an increase in foraminal dimensions while deviating from the original path, resulting in incomplete instrumentation of the cemental wall surrounding the foramen [12].

Despite the controversial data, FE significantly influences endodontic protocols by altering several aspects of root canal treatment. By widening the apical foramen, dental professionals can improve the efficacy of both irrigation and debridement, ensuring that cleaning agents reach all areas of the canal and reduce bacterial presence more effectively [5]. This increased access facilitates the removal of infected tissues and debris, contributing to a more thorough disinfection process [5].

However, while this procedure allows for better cleaning and enhanced obturation outcomes, it also poses risks such as the potential extrusion of irrigants and filling materials beyond the apex, which could lead to periapical irritation and postoperative discomfort [7]. As a result, protocols must address balancing these benefits and risks, requiring precise technique and sometimes modifying the choice of instruments and materials used.

FE can also impact the healing dynamics of periapical tissues, as they may alter natural anatomy and recovery processes. Consequently, endodontists must tailor their approaches, possibly incorporating advanced imaging and a detailed understanding of individual tooth anatomy to optimize treatment outcomes without compromising patient comfort and safety. Thus, it is necessary to assess the impact of the FE on postoperative pain perception after controlled endodontic treatment.

A systematic review with meta-analysis investigated the efficacy of apical foramen enlargement in endodontic treatments in reducing microbial load and its impacts on postoperative symptoms. The authors observed a significant increase in postoperative pain during the first seven days. However, there was no significant difference in analgesic consumption, acute flare-ups, or edema [13].

The Wave One Gold (Dentsply Sirona, Charlotte, USA) is a reciprocating instrument system with a single-file M-wire alloy. The potential advantages of this system include reduced instrument fatigue, good canal centering, and elimination of contamination that occurs with single-use instruments [14]. The instrument was chosen due to its advantages related to the superior flexibility, allowing better adaptation to the canal curvatures and minimizing the risk of fractures [14]. This specific motion minimizes continuous pressure on the canal walls, thereby reducing the likelihood of periapical tissue irritation due to material extrusion [14]. Additionally, the design features and flexibility of the instrument facilitate efficient debris removal within the canal, enhancing the efficiency of endodontic treatment and potentially contributing to a more comfortable postoperative recovery [14].

To our knowledge, the incidence of postoperative pain after foraminal enlargement with a single reciprocating instrument in molars has not been extensively studied. Therefore, the purpose of this study was to clinically evaluate the impact of postoperative pain and analgesic consumption associated with single-session endodontic treatment using the WaveOne Gold system with or without foraminal enlargement in mandibular molars with necrosis and acute apical periodontitis.

The null hypothesis tested was that there would be no difference in the incidence of postoperative pain and the number of analgesics used after endodontic treatment between the two groups.

## Materials and methods

**Fig. 1:**
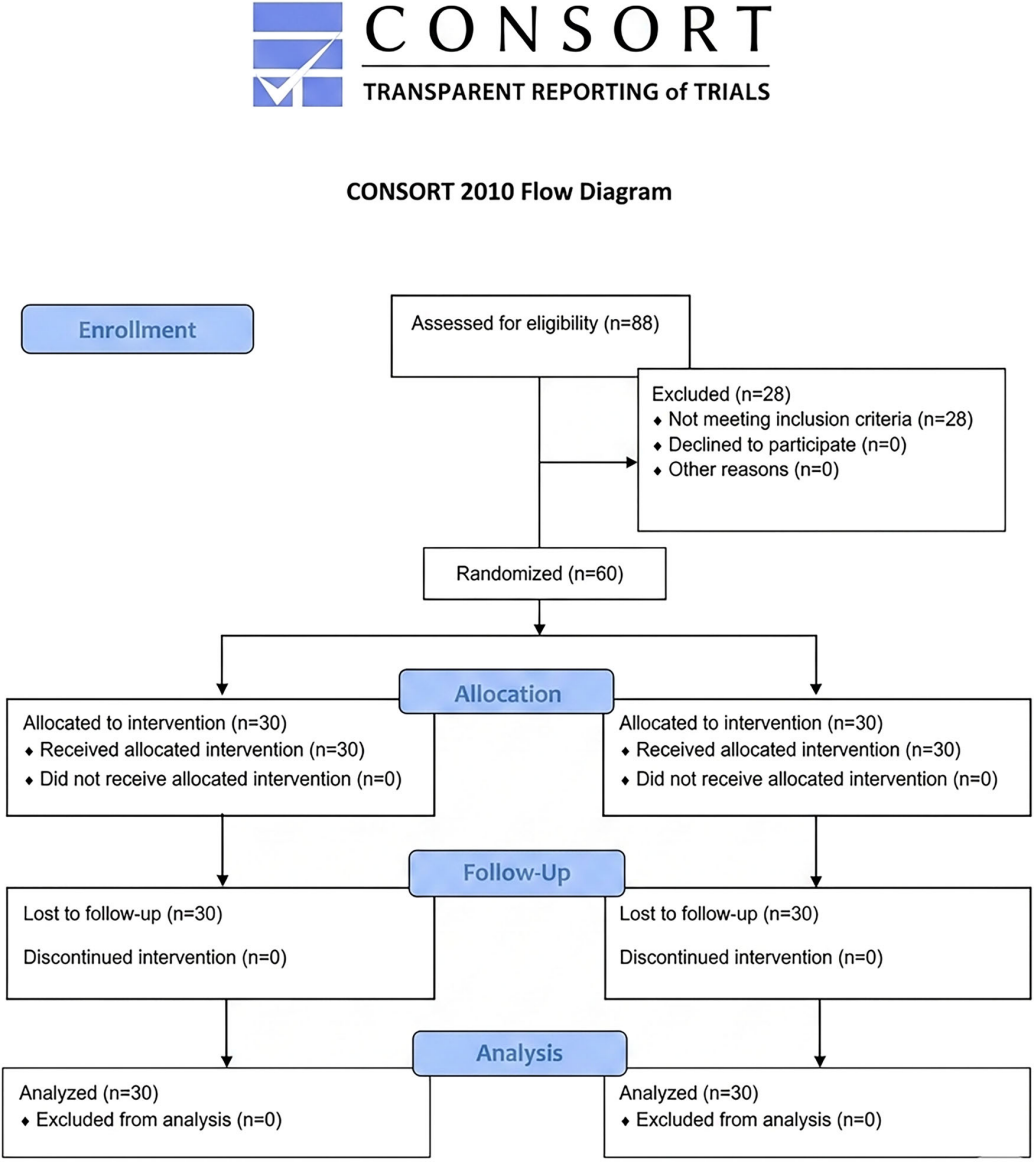
CONSORT (2010) flow chart showing the phases of the present study.

This study adhered to the Consolidated Standards of Reporting Trials (CONSORT) (Fig. 1). Ethical approval was obtained from the local research ethics committee (CAAE: 53140121.1.0000.5374). The sample size calculation followed the work of Yaylali et al. [3] using the *t*-test (G Power 3.1.9.4, Franz Faul, College of Kiel, Germany), with α = 0.05 and β = 0.80, effect size f = 0.75. The minimum sample size calculated for each group was 29 patients per group. Thirty patients were included in each experimental group (*n* = 30) and randomly assigned to the two groups. Patients were informed about the postoperative care, clinical and radiographic examinations, and signed the informed consent form. The randomization was performed blindly. Each patient selected one of two sealed and opaque envelopes containing the designation “with enlargement” or “without enlargement.” The patient was unaware of the contents. After the selection, only the operator opened the envelope and became aware of the technique to be used in the case. Each envelope was shuffled by an independent researcher before being offered to the patient for choice, ensuring the process remained unbiased. This method guaranteed that the allocation of techniques was random and concealed.

Inclusion criteria were mandibular molars with a diagnosis of pulp necrosis and asymptomatic periodontitis confirmed by a negative response to hot and cold tests and radiographic evidence of apical periodontitis. Exclusion criteria were pregnancy, treatment with antibiotics and analgesics in the last 1 month, diabetics and immunosuppressed patients. Only one tooth per patient was included in the study. All patients underwent thorough medical, dental, and clinical evaluations to exclude non-endodontic sources of orofacial pain that could confound postoperative pain assessment. This included screening for nasal/otorhinolaryngological disorders, periodontal conditions or food impaction, temporomandibular joint dysfunction, muscular pain, and other factors mimicking endodontic pain (e.g., sinusitis or septum syndrome). Only patients with symptoms consistent with pulp necrosis and asymptomatic apical periodontitis in the target mandibular molar—without clinical signs of these alternative conditions—were included, ensuring all recorded postoperative pain corresponded to a genuine endodontic origin linked to the treated tooth.

The endodontic treatment was performed in a single session by an endodontist who was familiar with the techniques, technologies, and materials used in the study. After a clinical examination and medical history, a thermal test was performed on the tooth to be treated and the corresponding homologous tooth to determine a response parameter/control. Postoperative periapical radiographs were performed on all patients to assess the quality of the obturation and to evaluate the potential extrusion of sealing material beyond the apex. No extrusion was observed in any of the cases. Additionally, the radiographs were carefully analyzed by two independent examiners to ensure accuracy in the evaluation process. Both examiners confirmed the consistent quality of the obturation and the absence of material extrusion. Once the diagnosis of pulp necrosis and/or apical periodontitis was established, the clinical endodontic treatment protocol was carried out. It should be noted that the patient did not know which group he was assigned to.

After anesthesia and coronal access, the canals were irrigated with 5 mL of 2.5% sodium hypochlorite (NaOCl) and explored with hand instruments type K #06, #08, or #10 (Dentsply Maillefer, Ballaigues, Switzerland), depending on the initial diameter of the foramen. This indicates an implicit assessment was performed to guide instrument selection for patency. Although not quantitatively measured as a distinct variable for postoperative pain analysis, this initial clinical consideration did not alter the observed pain outcomes.

The cervical and middle thirds of the root canals were prepared with a WaveOne Gold (Dentsply Sirona) with a diameter compatible with the anatomical canal, according to the manufacturer’s instructions. The working length (WL) was determined from the preoperative radiograph, and a Kerr file with a size and diameter compatible with the anatomy of the canal was inserted up to the apical foramen as determined with an electronic apex locator (Propex II, Dentsply Sirona; Ballaigues, Switzerland). For the FE group, WL was considered achieved when the file was set to the “0.0” position as indicated on the device display. The cursor on the device was set to the height of the occlusal margin selected as reference. The file was then removed from the canal, and the WL was measured with an endodontic ruler. For the WFE group, the WL measurement was performed following the same protocol but subtracting 1 mm from the “0.0” position shown on the device display.

In the WFE group (*n* = 30), the WL was set 1 mm short of the apical foramen, whereas in the group with FE (*n* = 30), the length was set at the apical foramen, as determined by randomization. The chemical-mechanical preparation of all teeth was performed with the same steps and protocols. The only difference was the WL at the apical foramen (enlargement of the apical constriction) or 1 mm in front of it (maintaining the apical constriction). All canals were instrumented with the WaveOne Gold system. The final diameter of each root canal was set according to the anatomical diameter of the root canal and coupled to the X-Smart Plus electric motor (Dentsply Sirona), with the reciprocating motion set according to the manufacturer’s recommendation. The instrument was used with back-and-forth movements with amplitudes of no more than 3 to 4 mm until the length set for each group was reached. After each insertion, the instrument was removed and cleaned with a gauze moistened with 70% alcohol.

The patency of the apical foramen was maintained throughout the chemical-mechanical preparation by inserting a #10 K-file 1 mm beyond the WL of each instrument.

The irrigation protocol used in all patients was identical: 2.5% NaOCl was renewed in a total volume of 30 mL throughout the chemical-mechanical preparation. A 5 mL disposable syringe with a 20×0.55 (Ultradent, Indaiatuba, Brazil) and a 30 G hypodermic needle (Ultradent) 3 mm from the WL was used. Final irrigation was performed by mechanical agitation with the EasyClean system (Bassi/Easy, Belo Horizonte, Brazil) coupled to the X-Smart Plus electric motor using 3 mL of 17% EDTA (ethylenediaminetetraacetic acid) for 1 min, activated in three 20-second cycles, advancing to 2 mm anterior to the WL. The canals were then rinsed with 6 mL of 2.5% NaOCl, also in three 20-second cycles. The canals were dried with absorbent paper (WaveOne System, Dentsply Sirona). The canals were filled with gutta-percha cones (WaveOne Gold System, Dentsply Sirona) corresponding to the last instrument used in the preparation, using AH Plus Sealer (Dentsply Sirona). After root canal filling, a 2-mm coltosol barrier (Vigodent, Rio de Janeiro, Brazil) was applied to seal the canal entrance with a subsequent composite restoration (Filtek™ Z250 XT, 3 M, São Paulo, Brazil), and the occlusion was checked and adjusted.

At the end of treatment, participants were given a visual analog scale (VAS) envelope and a chart on which they recorded their pain scores at 24 hours, 48 h, 72 h, and 7 days [15] and they were recommended to record the necessity of the analgesic use. VAS, represented by a 100-mm horizontal line; scores were analyzed on a 0–10 numeric scale for reporting, with accompanying verbal categories.

Patients were instructed to assign a value to their perceived pain based on the modified VAS. The presence or absence of pain was divided into four categories: no pain (0), mild pain (1–3), moderate pain (4–7), and severe pain (8–10). A single staff member, who was not the same as the person performing the endodontic procedure, kept in contact with the patients by telephone or message at 24-h, 48-h, 72-h, and 7-day [16] intervals to obtain information on pain perception and the amount of analgesic used, if necessary. The information was recorded on a worksheet. The analgesic prescribed was ibuprofen 600 mg every 6 hs.

### Statistical analysis

The data collected from the experimental groups were analyzed using IBM SPSS software (version 26.0, IBM Corporation, Armonk, New York, USA). The variables related to the characteristics of the participants in the two experimental groups were compared to assess and confirm comparability between the other variables of interest. Pearson’s chi-square test with and without Yates correction, Fisher’s exact test, and Student’s t-test were used for this purpose. Between-group comparisons of continuous VAS scores were performed using the Mann–Whitney U test due to zero-inflated/non-normal distributions. A significance level of 5% was used for all analyses.

## Results

The sixty patients enrolled in this study had an average age of 34 years. Baseline characteristics were statistically similar across groups; mean age was 33.97 ± 7.53 for the Foraminal Enlargement (FE) group and 33.60 ± 11.48 for the Without Foraminal Enlargement (WFE) group (*p* = 0.884). Sex distribution (e.g., 20 females in FE vs. 21 in WFE; *p* = 1.000) and treated tooth types (*p* = 0.502 for mandibular molars) also showed no significant differences, confirming sample compatibility (Table 1).

**Table 1:** Comparison between the experimental groups for the characteristics of the sample studied

Variable	Category	Group		P
		**FE**	**WFE**	
PAI index		1	1	
Age		33.97 ( ± 7.53)	33.60 ( ± 11.48)	0.884*
Sex	Female	20 (48.8%)	21 (51.2%)	1.000**
	Male	10 (52.6%)	9 (47.4%)	
Tooth	36	9 (56.2%)	7 (43.8%)	0.502***
	37	4 (36.4%)	7 (63.6%)	
	46	12 (60.0%)	8 (40.0%)	
	47	5 (38.5%)	8 (61.5%)	

FE = Experimental group treated with foraminal enlargement; WFE Experimental group treated without foraminal enlargement.

Significance level=5%

*Student’s *t*-test; **Pearson’s chi-square test; ***Pearson’s chi-square test with Yates’ correction.

At 24 h, 6 FE participants and 2 WFE participants reported pain (*p* = 0.254). The mean VAS score at 24 h was 0.77 ± 1.74 in the FE group and 0.20 ± 0.85 in the WFE group (Mann–Whitney *U*, *p* = 0.121). At 48 h, 2 FE and 1 WFE participants reported pain (*p* = 1.000); mean VAS scores were 0.10 ± 0.40 (FE) and 0.03 ± 0.18 (WFE) (*p* = 0.557). At 72 h and 7 days, no pain was reported, indicating resolution of postoperative pain (Tables 2, 3).

**Table 2:** Continuous VAS pain scores at each postoperative time point

Time point	Group	Mean ± SD	n with pain	*p*-value†
24 h	FE	0.77 ± 1.74	6/30	0.121
	WFE	0.20 ± 0.85	2/30	
48 h	FE	0.10 ± 0.40	2/30	0.557
	WFE	0.03 ± 0.18	1/30	

Patients recorded pain on a 100-mm VAS; scores were analyzed on a 0–10 numeric scale for reporting; zeros included for all randomized participants.

*FE* foraminal enlargement, *WFE* without foraminal enlargement.

†Between-group comparison using Mann–Whitney U test (VAS data zero-inflated/non-normal).

**Table 3:** Comparison of pain scores between the experimental groups

Variable	Category	Group		*P*
		FE	WFE	
Pain reported 24 h	Yes	6	2	0.254*
	No	24	28	
Pain reported 48 h	Yes	2	1	1.000*
	No	28	29	
Pain reported 72 h	Yes	-	-	-
	No	30	30	
Pain reported 7 days	Yes	-	-	-
	No	30	30	

FE = Experimental group treated with foraminal enlargement, WFE Experimental group treated without foraminal enlargement.

*Fisher’s exact test; Significance level=5%.

Postoperative pain, when present, was predominantly mild to moderate in both groups and occurred mainly within the first 24–48 h. Severe pain was rare and limited to the early postoperative period in the FE group. No severe pain was observed beyond 48 h, and pain resolved by 72 h in both groups. There was no significant between-group difference in pain intensity distribution at 24 h or 48 h (*p* > 0.05) (Table 4).

**Table 4:** Comparison of Pain intensity scores between the experimental groups

Variable	Category	Group			*P*
		FE	WFE		
Pain intensity 24 h	Mild	1	1	0.321*	
	Moderate	2	1		
	Severe	3	0		
Pain intensity 48 h	Mild	1	0	1.000*	
	Moderate	1	1		
	Severe	0	0		

FE = Experimental group treated with foraminal enlargement; WFE Experimental group treated without foraminal enlargement.

*Fisher’s exact test; Significance level=5%.

Analgesic use was also similar: 4 FE and 1 WFE participants took medication at 24 h (*p* = 0.353). The mean number of tablets taken at 24 h was 1.25 for FE and 1.00 for WFE (*p* = 0.685). At 48 h, only 1 FE participant took 1 tablet. These results indicate no statistically significant difference in postoperative pain incidence or analgesic consumption between the groups (Table 5).

**Table 5:** Analgesic intake frequencies

Variable	Category	Group		*P*
		FE	WFE	
Medication 24 h	Yes	4	1	0.353*
	No	26	29	
Medication 72 h	Yes	1	0	1.000*
	No	29	30	
Number of tablets	24 h	1.25^#^	1.00	0.685***
	48 h	1.00^#^	0	-

FE = Experimental group treated with foraminal enlargement; WFE Experimental group treated without foraminal enlargement.

*Fisher’s exact test; ***Student’s *t*-test. Significance level=5%. ^#^Values represent mean number of tablets taken among those who used analgesics.

## Discussion

This study investigated the effect of foraminal enlargement on postoperative pain in mandibular molars with necrotic pulp and apical periodontitis. The results of this study showed that there was no statistically significant difference in pain perception and the number of tablets taken for pain relief between the experimental groups. Therefore, the null hypothesis was accepted.

The mandibular first molar is often associated with a higher incidence of postoperative pain following endodontic treatment. Studies indicate that anatomical complexity and frequent use during mastication contribute to this relationship. According to the previous studies, these teeth are frequently reported as causing more discomfort after treatment due to their anatomy and high level of treatment complexity [17, 18].

Although the lower molars are multirooted teeth, the present results should be extrapolated with caution to other multi-rooted teeth because each tooth group may present its own anatomical complexity and varying canal structures, which can influence treatment outcomes and pain experiences. Additionally, the differences in procedural challenges and potential for unnoticed canal remnants can lead to variability in postoperative response.

FE is a technique used to prepare the cemental canal walls at the level of the apical foramen in order to remove the biofilm [13]. While the existing literature has examined non-instrumented areas of the dentin walls after mechanical preparation, quantitative evaluation of the extent of patent natural transport in the cement canal at the level of the apical foramen is limited. Most studies primarily evaluate the effects of FE qualitatively by considering factors such as deformations and deviations measured by scores, or they focus on the enlargement and morphology of the foramen [10, 19, 20].

A recent study evaluated the area, circumference, root canal transport, and centering index of preparations at 1, 3, and 5 mm from the foramen as well as non-instrumented walls. They used mesial roots of mandibular molars of the foramen only after patency, foraminal debridement, and FE with manual or reciprocal instrumentation. They conclude that mechanical preparation at the foramen resulted in FE and transportation, and could not contact all root canal walls delimiting the foramen.

Despite the above considerations, apical enlargement is a widely used protocol in necrotic cases due to its antimicrobial advantages over the cemental bacterial flora [13, 21]. Research studies have shown that intentional FE contributes to the healing of chronic periapical lesions [4, 22]. However, experts agree that maintaining the original position and dimensions of the apical foramen can help to prevent the inadvertent extrusion of irrigants, debris, and filling material [23]. In addition, this approach promotes effective sealing of the root canal as it allows better adaptation and compaction of the filling material in the apical region [24].

The enlargement of the foraminal area in endodontic practice carries significant implications for clinical outcomes and procedural protocols. It enhances the potential for thorough debridement and effective irrigation, improving the overall success of disinfection efforts within the root canal [25]. However, it also raises the risk of material extrusion and irritation in the periapical tissues, necessitating precision in instrumentation and careful management to mitigate postoperative discomfort [25, 26]. This requires practitioners to adapt their techniques, possibly employing advanced imaging and a nuanced understanding of tooth anatomy to achieve optimal results [25]. One of the main challenges of this study was the subjectivity of pain and its measurement. This makes clinical studies linking the occurrence of pain to possible causes extremely difficult [27]. The VAS and the follow-up protocol model were used to assess the results. It is a simple, efficient, easy-to-understand, and reliable model. It has been described as one of the most used methods in pain research [25, 26, 28]. Postoperative pain presents significant challenges in the objective quantification of pain assessment methods. The Visual Analog Scale (VAS) remains a prevalent tool in endodontic research due to its continuous nature, offering superior sensitivity for detecting subtle changes in pain intensity over time, which facilitates robust statistical analysis. The present study used a modified VAS incorporating both a 100 mm line and verbal categories, recognized for being “simple, efficient, easy to understand and reliable” (Zamparini et al., 2024). However, VAS methods are subject to individual perception and potential recall bias, a limitation acknowledged in the present study. Numeric Rating Scales (NRS), in contrast, provide a simpler, discrete numerical option, highly valued for rapid administration and verbal communication, as explored by Eyüboğlu et al. (2023) regarding clinical examination reliability. Yet, NRS offers reduced granularity compared to VAS, potentially limiting its ability to capture fine distinctions in pain (Arias et al., 2013).

Verbal Categorical Scales (VCS), while universally comprehensible and quick to administer, as partially integrated into the study’s modified VAS with descriptors like “nonexistent, mild, moderate, or severe”, suffer from the lowest sensitivity. This qualitative nature limits their capacity to detect nuanced pain fluctuations and can introduce variability due to subjective interpretation of terms, potentially affecting the comparability of findings (Zamparini et al., 2024). The choice of assessment tool critically influences reported pain incidence and intensity, underscoring why transparent methodology is vital for comparing studies, such as those investigating postoperative pain prevalence (Sabino-Silva et al., 2023) or factors influencing pain (Prasad et al., 2024; Sathorn et al., 2008). By employing a hybrid VAS approach, the present study aimed to balance quantitative precision with clear categorical descriptors, thereby allowing for a comprehensive evaluation of the observation that no severe pain was reported in their cohort, contributing valuable insights into the methodologies employed in endodontic pain research.

The occurrence of exacerbated pain after endodontic treatment is clinically relevant, well-defined [29] and affects the patient’s quality of life to such an extent that additional, unscheduled visits are required [30]. Previous studies have reported low rates of occurrence of severe postoperative pain: 3% [31] 1.9% [5] 0% [6, 16, 32].

In this study, no patient had severe pain requiring additional visits during the follow-up period. In contrast to this study, some studies [2, 3, 32] have shown a higher incidence of postoperative pain in patients undergoing endodontic treatment with FE. The type of methodology and instruments used could explain the differences in the results. Patients reported moderate and severe pain for up to four days after two sessions of endodontic treatment with hand files, with intracanal medications administered between sessions, without irrigant agitation [33]. The use of hand files has contributed to greater debris extrusion compared to rotary and reciprocating instruments [34]. The use of the XP-Endo Finisher after endodontic treatment with foraminal enlargement caused moderate postoperative pain [35].

In this study, 2.5% NaOCl was used for root canal irrigation, as this is the most used irrigation solution worldwide. This differs from another study in which 2% chlorhexidine was used, and a considerable success rate was achieved [36]. Sodium hypochlorite, frequently used as an irrigant during endodontic treatment, plays a crucial role due to its antimicrobial and tissue-dissolving properties [37]. However, studies have indicated that sodium hypochlorite, while effective in eliminating microorganisms, can cause tissue irritation, resulting in discomfort after the procedure due to chemical aggression, resulting in irritation of periapical tissues and consequently an inflammatory response that causes pain [38, 39]. On the other hand, lower concentrations, though potentially less effective in disinfecting the canals, can reduce the risk of postoperative pain by minimizing aggression to periradicular tissues [40].

Regarding postoperative pain, systematic reviews have observed contradictory results [39, 40]. In 2023, another study found that the prevalence of pain was 45% when using NaOCl at a low concentration and 39% when at a high concentration, with pain decreasing 24 and 48 h after treatment. The prevalence of medication use was 9% with low concentration and 15% with high concentration. In another more recent review, the authors compared NaOCl 3% and NaOCl 5% and reported less pain for 3% after 24 and 48 h, as well as a reduced need for analgesics, compared to the high concentration [39].

The chlorhexidine gluconate is widely used as an irrigant in root canals due to its effective antimicrobial properties [41]. It works by eliminating resistant bacteria, contributing to the decontamination of the canals during endodontic treatment. Additionally, as it has a low potential for tissue irritation, it can help minimize postoperative pain [41, 42].

This study also differs from the results of another study [32] which used a similar methodology but differed in the use of single-rooted teeth and ultrasonic agitation of the irrigation solution. The results showed that pain was reported more frequently in the FE group in the first 24 h, but as in the present study, there was no statistically significant difference in the use of analgesics. In another study [16]. There was also no statistically significant difference in the incidence of postoperative pain in patients who underwent root canal instrumentation with one rotary and two reciprocating systems.

Studies on molars using reciprocating systems to assess the effects of foraminal enlargement are scarce in the literature to compare the results, which makes the present study relevant and its results a contribution to the endodontic literature. A previous study on molars showed that postoperative pain was most severe in the first two days after FE with rotary systems [3]. These differences may be related to the different instrument cross-sections, tip design, instrument taper, flexibility, alloy type, number of instruments used, and cutting kinematics [43].

Regarding the use of analgesics, the results confirm the findings of several studies, which showed that the FE of the apical foramen did not influence the number of tablets used [2, 32, 33].

However, it is important to point out the subjectivity of the decision for or against analgesic administration. The limitations of the present study should be considered. The data on root canal sealer extrusion and its relationship to pain perception were not analyzed. It should also be noted that the different subjective pain thresholds of the patients may influence the values assigned in the VAS.

The sealer extrusion during endodontic treatment can significantly impact postoperative pain, a common concern among patients undergoing root canal therapy. This phenomenon occurs when the sealer is unintentionally pushed beyond the apex of the tooth into the surrounding tissues. The resultant irritation can provoke an inflammatory response, leading to varying degrees of postoperative discomfort [25]. Factors contributing to sealer extrusion include aggressive obturation techniques and anatomical complexities, such as large apical foramina or root resorption [28]. To mitigate this risk, endodontists often employ advanced imaging techniques like digital radiography or cone-beam computed tomography (CBCT) to better understand the root canal anatomy and ensure precise sealer placement [26].

Choosing the right type of sealer, one that causes minimal tissue irritation, also plays a crucial role in managing pain. While modern sealers are designed to be biocompatible, the body’s reaction to even slight extrusion can still lead to sensitivity or mild discomfort [28]. Patient education about potential sensations following treatment is essential in managing expectations and ensuring compliance with postoperative care instructions, which can involve the use of analgesics or anti-inflammatory medications to manage pain as healing progresses [25, 28].

Bias in the results was minimized as participants were blinded throughout the study and at follow-up. The variables characterizing the sample were similar in the two groups studied, especially in relation to the treated tooth, as the study was conducted on mandibular molars only and by a single endodontist. Another potential bias in reporting endodontic postoperative pain includes recall bias, where patients may not accurately remember their pain levels over time, and response bias, where patients might underreport pain due to social desirability or expectations [38, 42]. Additionally, measurement bias can occur if inconsistent or non-standardized pain assessment tools are used across studies or clinical settings, potentially skewing results [25, 28].

Studies with contradictory results can be found in the literature. There is a need for a systematic review summarizing the results of these types of studies. There is also a need for studies on FE and its influence on periapical tissue healing in necrotic teeth and teeth with apical periodontitis [19] as well as observational cohort studies to elucidate the long-term effects of FE. Additional metrics for evaluating treatment success include Patient-Reported Outcome Measures (PROMs) for assessing quality of life improvements, quantitative microbial analysis to ensure effective bacterial reduction, and biomarker evaluation in saliva or serum to monitor healing. Further, metrics like function restoration and long-term tooth survival rates provide critical insights into the structural and operational success of the treatment.

Recent advances in endodontic technologies have significantly mitigated postoperative pain, primarily through enhanced imaging techniques, like cone-beam computed tomography (CBCT), which allow for precise diagnosis and treatment planning [39]. Additionally, the development of more effective irrigation systems and biocompatible sealers has improved the disinfection process and healing outcomes [28]. These innovations not only reduce pain but also increase the overall success rate of endodontic procedures by allowing clinicians to address complex cases with greater accuracy and efficiency [13].

Future research on foraminal enlargement should be performed with new techniques in order to evaluate the long-term effects on both periapical healing and potential risks, such as root structure weakening, across diverse patient populations. Studies could also explore advanced imaging technologies to better understand the biological and mechanical implications of foraminal manipulation during endodontic treatment.

## Conclusion

The results of this study show that although the working lengths during endodontic treatment were different in the two groups studied, the incidence of postoperative pain and the analgesic medication required were similar in the two experimental groups. Additionally, routine follow-ups for monitoring healing progress and addressing any complications would enhance the patient’s comfort after the endodontic treatment.

### Ethics approval and consent to participate

This clinical trial adhered to the principles of the Declaration of Helsinki and followed the Consolidated Standards of Reporting Trials (CONSORT) guidelines. Ethical approval was obtained from the Research Ethics Committee of Faculdade São Leopoldo Mandic (CAAE: 53140121.1.0000.5374). All participants provided written informed consent before enrollment in the study.

## Data Availability

The datasets generated and analyzed during the current study are available from the corresponding author upon reasonable request.
